# Study of Organic Solvent Tolerance and Increased Antibiotic Resistance Properties in *E. coli gyrA *Mutants

**Published:** 2012

**Authors:** Razieh Pourahmad Jaktaji, Rayhaneh Ebadi, Marzieh Karimi

**Affiliations:** a *Department of Genetics, Faculty of Sciences, University of Shahr-e-Kord, Shahr-e-Kord, Iran. *; b *Institute of Biotechnology, University of Shahr-e-Kord, Iran.*

**Keywords:** GyrA mutants, Organic solvent tolerance, Tetracycline resistant mutants, MarR mutation

## Abstract

Ciprofloxacin is one of the most widely used antibiotics for the treatment of several infections caused by Gram-negative bacteria, like *E. coli*. Changes in *gyrA*, encoding GyrA subunit of DNA gyrase, cause the resistance to ciprofloxacin. Some ciprofloxacin resistant *gyrA* mutants acquired constitutive expression of *marRAB* operon due to the gaining mutations in *marR*, a repressor of this operon. This leads to the expression of a multidrug resistance phenotype and high organic solvent tolerance. Thus, this study was aimed to provide more information on extra mechanisms of resistance in *gyrA* mutants with different ciprofloxacin MICs. For this purpose, the tolerance of organic solvent, resistance to tetracycline and presence of possible mutation in *marOR* were investigated in 10 *gyrA* mutants. Results showed that most of *gyrA* mutants behaved like MG1655, control strain, but 3 out of 10 were slightly more resistant to tetracycline than MG1655 and had better growth on hexane. Among three mutants, two possess a mutation in *marOR*. In conclusion, the generation of mutation in *marOR* is not enough by itself to produce the multidrug resistance phenotype and complete activation of AcrAB-TolC.

## Introduction

Ciprofloxacin is one of the most widely-used antibiotics for the treatment of infections, such as urinary tract infections, caused by Gram-negative bacteria, like *E. coli* (1, 2). However, there is growing evidence which shows that the frequency of resistance to ciprofloxacin among *E. coli* isolates is rising yearly all over the world (3, 4, 5). This resistance has been linked to the chromosomal mutations that cause either changes in *gyrA* and *gyrB*, the main target of antibiotic or decreases the accumulation of drug inside the bacteria. However, in higher quinolone-resistant mutants, both of these are the case (6).

In the previous study, we described *E. coli* ciprofloxacin resistant mutants that have mutations in *gyrA* gene (7). This gene, along with *gyrB* gene, encodes DNA gyrase subunits (7, 8). This enzyme catalyzes the negative supercoiling of DNA required for chromosome replication, transcription and recombination (8, 9).

**Figure 1:**
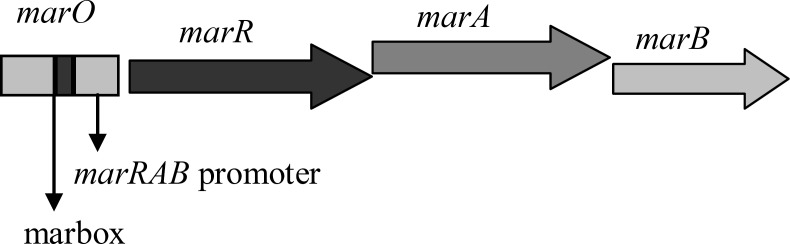
Schematic representation of *marRAB* operon in *E. coli*. *marO* harboring promoter and marbox. Simplified and adapted from Cohen *et al*., 1993 (14).

These* gyrA* mutants have different MICs against ciprofloxacin (7). Thus, it is possible that some with higher level of resistance may have extra mutations that decrease the amount of ciprofloxacin inside the cells (6). This may result from the activation of endogenous transmembrane efflux pump, AcrAB-TolC (10). This is a multidrug efflux pump consists of three components, including AcrB, the inner membrane transporter protein, TolC, the outer membrane channel, and AcrA, the periplasmic membrane fusion protein (10). This pump is activated through MarA. MarA is a transcriptional activator which activates its own transcription and that of a large number of *mar* regulon genes, including *AcrAB*,* tolC* and *micF*, the repressor of OmpF, via binding to special sequences called marboxes that are located in the upstream of promoters for the target genes (11, 12). This protein is produced by *marA*, a member of *marRAB* operon (Figure 1). *MarR* is another member of operon which encodes a repressor protein. MarR controls the intracellular levels of MarA through binding to two certain sequences in *marO*, the regulatory region of operon. One is between -35 and -10 sequences and the other is just near the initiation codon (13). Thus, mutations that inactivate *marR* can increase the expression of *marA* gene and thereby can enhance the ejection of ciprofloxacin and decrease the entry of some other antibiotics, such as ampicillin, chloramphenicol, and tetracycline. This happens first, through the increase in the activity of AcrAB-TolC pump and second, via the decreases in the synthesis of porin proteins, OmpF, which are used as a route of cell entry for above mentioned antibiotics. It was proposed that *mar* regulon is associated with clinical antibiotic resistance and treatment failure (14). This regulon is also related to the organic solvent tolerance. The correlation between the high levels of organic solvent tolerance and low levels of resistance to some antibiotics as mentioned above has already been explained (15, 16).

**Table 1:** Ciprofloxacin susceptibility of control strain and its derived mutants

**Strain/mutant**	**MIC (ng/mL)**
**MG1655 wild type**	35
**W10-W11**	62.5
**W25-W26**	75
**W44-W45**	125
**W47-W48**	312
**W49-W50**	625

It was reported that some of the quinolone-resistant *E. coli* isolations acquired constitutive expression of *marRAB* operon due to the occurrence of mutations in *marR* (17). To gain more information on extra mechanisms of resistance in *gyrA* mutants, they were examined for acquirement of high organic solvent tolerance, resistance to tetracycline and mutations in *marR* gene.

## Experimental

*Antimicrobial agent and chemicals*

Tetracycline hydrochloride (Tc) was obtained from Sigma. Stock solution was 4 mg/mL. Organic solvents used for this study were *n*-hexane (Merck) and cyclohexane (Merck).

*Bacterial strain and mutants*

MG1655 was parent strain. *GyrA* mutants isolated in previous work (7) are listed in Table 1.

*Media*

LB broth (Merck) was used to prepare LBGMg agar medium, containing 0.1% glucose, 10 mM MgSO_4_ and 1.5% agar other than LB.

*Organic solvent tolerance assay*

Serial dilutions were prepared from fresh cultures of strains and mutants in 0.9% NaCl and 5 µL of each dilution spotted on a solid LBGMg medium as described previously (15). The surface of the medium was overlaid with an organic solvent and incubated at 37°C for 24 h. Then, the number of colonies per spot was counted on each plate.

**Table 2:** Organic solvent tolerance of control strains and *gyrA *mutants.

**Stran/mutant**	**No. of bacteria spotted**				
	**Without solvent**	**H** ^a^	**H-CH** ^b^ ** (3:1)**	**H-CH** ^b^ ** (1:1)**	**H-CH** ^b^ ** (1:3)**
**MG1655**	30*10^6^	19*10^4^	0	0	0
**W10-W11**	26*10^6^	19*10^4^	0	0	0
**W25-W26**	22*10^6^	6*10^5^	0	0	0
**W44-W45**	20*10^6^	19*10^4^	0	0	0
**W47-W48**	20*10^6^	21*10^4^	0	0	0
**W49**	17*10^6^	5*10^5^	0	0	0
**W50**	19*10^6^	21*10^4^	0	0	0

^a^H, hexane, ^b^H-CH mixture of hexane and cyclohexane.

*Antibiotic susceptibility test*

As described in previous study (7), MICs of Tc for control strain, MG1655, and *gyrA *mutants were determined using broth dilution method (18). Different concentrations of Tc ranging from 0.125 µg/mL to 5 µg/mL, were used. MICs for control strain and *gyrA* mutants were determined in three independent experiments.

*PCR amplification and DNA sequencing*

A single colony from each strain and mutants grown on LB agar was used as a template for PCR reaction as described previously (7). Primers used for PCR amplification and DNA sequencing were forward primer 5`-GGTGGTTGTTATCCTGTGTA-3` and reverse primer 5`-CGGCAGGACTTTCTTAAGC-3`. PCR products (700 bp in size) which contained part of *marO* and the entire *marR* gene were sequenced.

## Results and Discussion

Ten *gyrA* mutants and their parent strain MG1655 were assessed for organic solvent tolerance. Results are shown in Table 2. As can be seen from this table, MG1655 can grow in presence of hexane, but not cyclohexane. This is consistent with the previous result for this strain (15). All mutants derived from this strain also show nearly the same results. Some mutants had slightly better growth on hexane.

To assess whether *gyrA* mutants may acquire resistance to other antibiotics, the MIC of Tc for control strain and *gyrA* mutants were measured. For MG1655, MIC was 3 µg/mL (Table 2). This is consistent with previous data (19). MICs of *gyrA* mutants were nearly the same as that of MG1655, except some seemed to show better growth at 3 µg/mL tetracycline and their MICs were 4 µg/mL (Table 2). Collectively, these results suggest that *gyrA* mutants may either not acquire a mutation in *marR* gene, or some of them acquire a mutation, but cannot cause high level of organic solvent tolerance and partial resistance to other antibiotics at once and need to be induced in the presence of inducers, such as tetracycline (19).

**Table 3:** Tetracycline susceptibility of control strain and its derived mutants.

**Strain/mutant**	MIC* (μg/mL)
**MG1655 wild type**	3
**W10-W11**	3
**W25-W26**	4
**W44-W45**	3
**W47-W48**	3
**W49**	4
**W50**	3

*Data are mean of at least two independent experiments.

**Figure 2:**
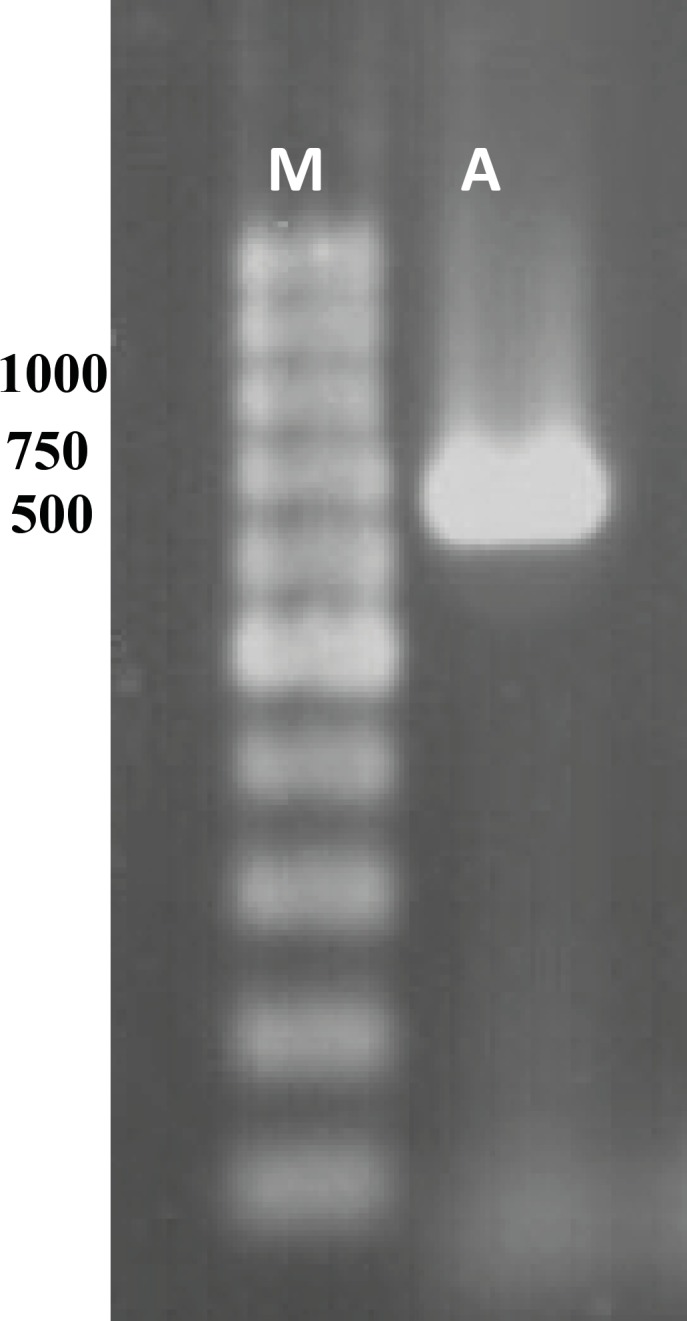
Gel analysis of PCR product. Lane M and A contain 1 Kb DNA ladder and PCR product, respectively.

**Figure 3:**
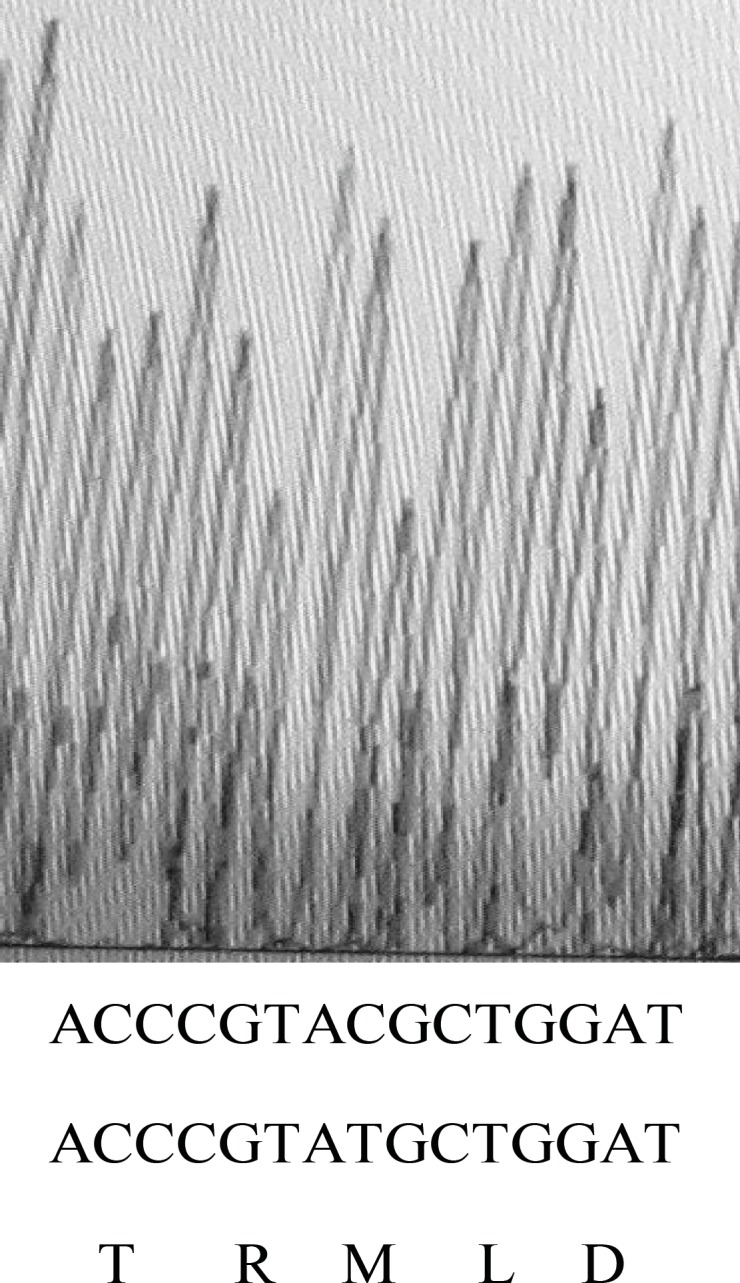
Sequence output from PCR product of *gyrA* mutant using forward and reverse primers. On the bottom of graph, the first and second rows of nucleotide sequences belong to mutant and wild type strain, respectively. The third line shows the amino acid sequence of that region in wild type strain.

**Figure 4:**
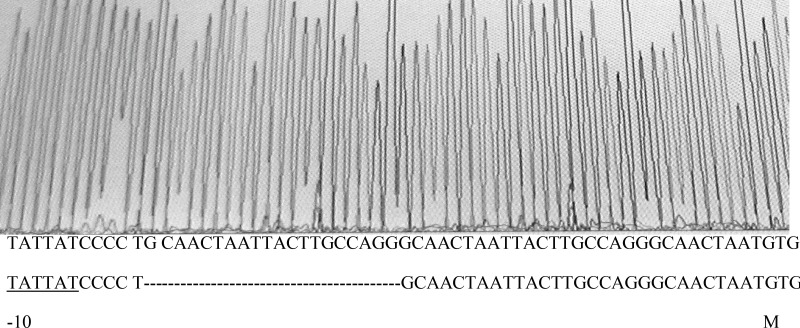
Sequence output from PCR product of *gyrA* mutant using forward and reverse primers. On the bottom of graph, the first and second nucleotide sequences belong to mutant and wild type strain, respectively. The -10 region of promoter, duplicated sequence and start codon are shown

To verify this *marR* gene and its upstream region, *marO* in MG1655 and its 10 *gyrA* mutants were amplified. Figure 2 shows the result of gel electrophoresis of the *marOR* PCR product of MG1655. The same results were obtained for all *gyrA* mutants. Then, the PCR products were sequenced using forward and reverse primer. Finally, the sequences were compared with the published *marR* sequence of MG1655 strain, using EBI bioinformatics program for nucleotide pair wise alignment. MG1655 showed the complete match with previous published sequence for *marR* (data not shown). Moreover, 7 *gyrA* mutants also showed the same result as MG1655. Their Marbox and promoter site were also intact. This suggests that their higher ciprofloxacin MICs may not be associated to the induction of drug efflux. However, 2 mutants with higher Tetracycline MIC, had mutations in *marOR*. One contained a T→C change at nucleotide position 221 in coding region of *marR* that alters methionine-74 to threonine (Figure 3), but its marbox and promoter site was without change. The other mutant harbors, a 20-base pair tandem duplication of a sequence (GCAACTAATTACTTGCCAGG) started 6-base pair downstream from the 10-position of promoter site (Figure 4). This type of mutation was described before (14). However, its marbox was intact. The last mutant with higher tetracycline MIC did not possess a mutation in *marOR*. This suggests that the reason of slightly different tetracycline MIC may be the occurrence of mutation in a gene that encodes a suppressor for the pump.

In the previous study, ciprofloxacin-resistant mutants which possess mutations in *gyrA* were isolated (7). These *gyrA* mutants had different levels of resistance to ciprofloxacin. It was explained that one reason for this difference is due to the decreased levels of drug accumulation (6). The intracellular amount of antibiotic can be regulated through a efflux pump, AcrAB-TolC (10). The activation of this pump is associated with the presence of MarA, the transcription activator protein. The gene that encoded this protein is located in *marRAB* operon, consisting *marR*, *marA* and *marB* genes. This operon is normally inactivated through the product of *marR* gene, MarR. The inactivation of MarR is caused through the mutations in *marR* (17). This leads to the expression of a multidrug resistance phenotype (MDR). This phenotype is also associated to the high level of organic solvent tolerance (15, 16). Therefore, in this study, ciprofloxacin-resistant mutants with different MICs were examined for the tolerance of organic solvents, resistance to tetracycline and presence of *marR* mutation.

The obtained results revealed that none of the *gyrA* mutants even those with higher ciprofloxacin MICs possess the MDR phenotype de novo, but some *gyrA* mutants may gain this phenotype gradually following the exposure to an inducer of *marRAB* operon, such as tetracycline. This was revealed through the discovery that 3 out of 10 *gyrA* mutants are slightly more tolerant to hexane and resistant to tetracycline compared to MG1655.

Our finding that 7 *gyrA* mutants are the same as MG1655 for sensitivity to tetracycline, tolerance of organic solvent and devoid of *marOR* mutation, implies that the reason for their higher ciprofloxacin MICs in comparison with MG1655 may be the presence of mutations in *gyrB*, *parC* or *parE* (6). The last two genes encode the subunits of topoisomerase IV, the minor target of fluoroquinolone antibiotics in *E. coli* (6).

Moreover, an *E. coli *mutant (OST3408) was found by Asako that is tolerant not only to hexane, but also to cycloheexane (15). Most of *E. coli* strains are sensitive to cyclohexane. This mutant contains a substitution of serine for arginine at position 73 in the coding region of marR. The MarR protein has been shown to contain the helix-turn-helix motif started from the 60^th^ amino acid codon and extended to the 80^th^ one (20). This motif is necessary for the binding of repressor to the promoter site. However, we found that a T→C mutation in marR that causes a substitution of threonine for metionine at position 74 does not confer tolerance to cyclohexane. This implies that both the position of amino acid and the type of substitutions in amino acid sequence of this motif are important on functionality of repressor. On the other hand, the alteration of the repressor binding sites interferes with repressor activity. We found a *marR* mutation with altered repressor binding sites that has already been discovered (14). It was found that this kind of mutation increases the activity of *marRAB* operon (14).

Moreover, mutations in *acrR* gene, encoding the repressor of AcrAB-TolC pump called AcrR, increase the activity of the pump (21). This fact that one of the three slightly resistant mutants did not possess a mutation in *marOR,* implies that this *gyrA* mutant may acquire a mutation in *acrR* gene.

Furthermore, highly organic solvent tolerant mutants gain mutations both in *marOR* and *acrR* (22). Therefore, it is possible that our *gyrA marR* double mutants are not resistant to cyclohexane due to their lack of a mutation in *acrR*.
